# Frequency of Self-reported Unpleasant Events and Harm in a Mindfulness-Based Program in Two General Population Samples

**DOI:** 10.1007/s12671-020-01547-8

**Published:** 2020-12-02

**Authors:** Ruth Baer, Catherine Crane, Jesus Montero-Marin, Alice Phillips, Laura Taylor, Alice Tickell, Willem Kuyken, The MYRIAD team

**Affiliations:** Department of Psychiatry, Warneford Hospital, University of Oxford, Oxford, OX3 7JX UK

**Keywords:** Mindfulness-based program, Secondary teachers, University students, Adverse effects, Side effects

## Abstract

**Objectives:**

Evidence-based mindfulness programs have well-established benefits, but the potential for harmful effects is understudied. We explored the frequency and severity of unpleasant experiences and harm in two nonclinical samples participating in an adaptation of mindfulness-based cognitive therapy (MBCT) for the general population.

**Methods:**

Study 1 included 84 schoolteachers; study 2 included 74 university students. Both studies were uncontrolled. Participants completed self-report questionnaires about psychological symptoms before and after the 8-week mindfulness course. After the course, they responded to a survey designed for this study that included Likert ratings and free-text questions about unpleasant experiences and harm. All data were collected online.

**Results:**

In both samples, about two-thirds of participants reported unpleasant experiences associated with mindfulness practice during the course. Most participants (85–92%) rated these experiences as not at all or somewhat upsetting; some indicated that difficult experiences led to important learning or were beneficial in some way. The proportion of participants reporting harm from the mindfulness course ranged from 3 to 7%. The proportion showing reliable deterioration on symptom questionnaires ranged from 2 to 7%. Those reporting harm and those showing reliable deterioration on questionnaires were largely separate subgroups; only one participant fell in both.

**Conclusions:**

Findings highlight the need for mindfulness teachers to manage expectations about benefits and difficulties that may occur in mindfulness-based programs and to work skilfully with participants experiencing difficulties. Experiences of harm may not be captured by symptom questionnaires and should be explicitly assessed in other ways.

**Supplementary Information:**

The online version contains supplementary material available at 10.1007/s12671-020-01547-8.

The benefits of mindfulness-based programs (MBPs) for a wide range of populations and outcome variables are well supported in meta-analytic reviews (Dawson et al. 2019; Demarzo et al. 2015; Goldberg et al. 2018; Khoury et al. 2013). However, the integrity of MBPs relies not only on demonstrating benefits but also on the Hippocratic imperative to do no harm. Interest in whether MBPs might cause harm arises from several related lines of research (Baer et al. 2019). First, in the psychotherapy literature, the potential for harm, defined as sustained deterioration in a participant’s functioning that is attributable to the therapy (Dimidjian and Hollon 2010; Duggan et al. 2014), has been recognized for several decades (Bergin 1966) and studies consistently show that between 3 and 10% of psychotherapy clients get worse (Crawford et al. 2016; Hansen et al. 2002; Lambert 2013; Mohr 1995; Strupp et al. 1977). Evidence-based MBPs are commonly described as educational or skills training programs, rather than forms of psychotherapy, but are often used in clinical settings to reduce stress and psychological symptoms. It is therefore important to examine whether rates of deterioration in MBPs are comparable to those seen in psychotherapy. Second, MBPs are organized around the formal practice of mindfulness meditation using practices designed for contemporary mainstream settings but with roots in Buddhist traditions. Several uncontrolled descriptive studies have reported serious harm in practitioners of Buddhist forms of meditation such as Vipassana and Zen (Lindahl et al. 2017; see Baer et al. 2019, for a review). Study of whether similar effects might arise in evidence-based MBPs is essential. Third, it is widely recognized that any program or intervention powerful enough to have substantial benefits might also cause harm (Dimidjian and Hollon 2010) and that the study of treatment failure and negative outcomes can lead to improved treatment methods (Dimidjian and Hollon 2011; Mohr 1995).

The literature on harmful effects of MBPs is sparse. A few trials have reported deterioration on some variables in clinical and nonclinical samples, including stressed workers (Brooker et al. 2012), adolescents in a school setting (Johnson et al. 2016), and cancer patients (Reynolds et al. 2017). However, meta-analyses have concluded that most studies show beneficial effects of MBPs in these populations (Dunning et al. 2019; Lomas et al. 2019; Piet et al. 2012). In a review of adverse effects of MBPs, Baer et al. (2019) found that many papers do not report on harmful outcomes. In the minority of studies providing such data, increases in symptoms or the appearance of new symptoms occurred in 0–10.6% of participants. These effects were generally described as transitory, not clinically significant, or as opportunities to learn to work skilfully with difficult symptoms (Banks et al. 2015), rather than as harmful. Reviews of randomized trials have shown that adverse effects, when reported, are no more common in MBPs than in control groups (Kuyken et al. 2016; Wong et al. 2018).

Overall, these findings suggest that the practice of mindfulness meditation in evidence-based MBPs can be uncomfortable and difficult, but that harm is probably rare. However, these findings must be interpreted cautiously for several reasons. First, adverse and harmful effects are often not reported in studies of psychological therapies broadly and MBP studies specifically. This may be due to the expectation that temporary increases in symptoms or discomfort are part of the therapeutic or skills training process (Peterson et al. 2013). Second, adverse effects are sometimes narrowly defined, drawing on definitions from medical research, such that general deterioration in mental state or the appearance of new psychological symptoms might not be included (Duggan et al. 2014). Finally, Duggan et al. (2014) noted that adverse events may not be spontaneously reported and will not come to light without systematic assessment methods. The current literature includes no established methods for asking participants in MBPs about harmful outcomes.

The purpose of this study, therefore, was to add to the literature on unpleasant experiences and harmful effects of evidence-based MBPs by posing a set of questions about such experiences to participants in 8-week group mindfulness programs. We hypothesized that reports of unpleasant experiences would be common, that many of these would be familiar to experienced mindfulness teachers (difficult thoughts, emotions, and bodily sensations; distress about not practicing mindfulness regularly or “correctly”), and that some of these unpleasant experiences would be seen as learning opportunities rather than as harmful. By providing open-ended opportunities to describe experiences of unpleasantness and harm, we explored whether unexpected and previously under-researched examples might come to light. We also explored relationships between unpleasant experiences and harm and scores on measures of psychological symptoms.

## Study 1

### Methods

#### Participants

Participants were schoolteachers participating in a larger study of mindfulness training in schools (see Crane et al. 2020, for full details). Teachers were eligible for the larger study if (a) they were willing to complete an 8-week personal mindfulness course in preparation for training to deliver a mindfulness curriculum to their pupils; (b) they had not completed a mindfulness course in the past year; and (c) they had not previously been trained to teach mindfulness to others. Schools were randomized to one of several training routes when a minimum of three teachers from that school had consented to take part. Here, we report data from participants who were randomized to a face-to-face 8-week personal mindfulness course delivered at their school, attended at least one session, and provided post-course feedback.

In total, 105 participants were randomized to the face-to-face mindfulness course. Of these, 19 were excluded from analysis because they did not complete the post-course measures. Fourteen of these 19 excluded participants completed less than 4 of the 8 mindfulness training sessions and the remaining 5 completed between 4 and 6 sessions. A majority of the participants who did not provide post-course measures withdrew from the study for logistical reasons or because they were unable to complete later aspects of the study protocol. Only one participant reported withdrawing because of not enjoying the mindfulness training. Two further participants were excluded because whilst they completed post-course questionnaires, they did not attend any of the mindfulness sessions.

The final sample of 84 participants included 68 women and 16 men ranging in age from 25 to 59 years (*M =* 38.00; *SD* = 9.70). Ninety-one percent identified as White British (*n* = 76); 1% as Black, African, or Caribbean (*n* = 1); 2% as Asian or Asian British (*n* = 2); 5% as White Other (*n* = 4); and 1% as Other (*n* = 1).

#### Procedure

Teachers completed a baseline assessment (T0) in autumn 2015 and began their personal mindfulness course in early 2016. Following completion of this course (T1), participants were sent a link to an online Qualtrics questionnaire for post-course measures. All training was delivered free of charge. Participants were compensated £25 in Amazon vouchers for completion of each set of study questionnaires. Schools were given £250 to spend on school resources at the end of the study.

The study was approved by the University of Oxford Medical Sciences Interdivisional Research Ethics Committee (20/03/2015; ref.: MS-IDREC-C1-2015-048). The study was also overseen by a Data Monitoring and Ethics Committee. The cluster-randomized feasibility study from which these data were drawn was registered at the ISRCTN (24/11/2015), prior to obtaining participant consent to randomization, 10.1186/ISRCTN18013311.

##### Mindfulness Program

The face-to-face mindfulness course was an adaptation of mindfulness-based cognitive therapy (MBCT; Segal et al. 2013) based on the book “Mindfulness: Finding Peace in a Frantic World” (M-FP; Williams and Penman 2011). M-FP was designed as an introduction to mindfulness training for the general population. It consists of eight weekly 90-min sessions and suggested home practice of 10–20 min per day. One 8-week course was delivered at each school for groups ranging from three to nine participants. Sessions occurred weekly (excluding school holidays) at times convenient for participants’ teaching schedules. Each M-FP course was delivered by a trained and experienced mindfulness teacher who met the good practice guidelines developed by the UK Network for Mindfulness-Based Teacher Training Organizations (http://mindfulnessteachersuk.org.uk/#guidelines) and had received further training and supervision. Participants were encouraged to read the relevant chapters of the book by Williams and Penman (2011) alongside their weekly sessions and were given a booklet to support their home practice. Participants were also given access to a publicly available app which accompanies the course and a CD or MP3 of the course material. Attendance data were collected for each participant.

Participant safety was addressed in several ways. Mindfulness teachers discussed contraindications with potential participants; none were advised not to take the course. Mindfulness teachers were asked to report to the research team any safeguarding issues that might arise (concerns of serious risk of harm to self or other); no such concerns were reported. Participants reporting psychological distress on questionnaires were managed within the study risk and safeguarding protocol.

#### Measures

For the larger study, participants completed a variety of measures of symptoms, wellbeing, and other outcomes. Findings for these measures are reported in separate papers (Crane et al. 2020; Montero-Marin et al. under review). For the present study, changes in anxiety and depression symptoms were examined so that rates of reliable improvement and deterioration could be compared with reports of unpleasant experiences and harm. 

Firstly, participants were asked to indicate sociodemographic data such as age, gender, and ethnicity. The Patient Health Questionnaire (PHQ-9; Kroenke et al. 2001) was used to measure depressive symptomatology. The PHQ-9 is a 9-item measure assessing depression severity in the preceding 2 weeks (e.g. “Thoughts that you would be better off dead or hurting yourself in some way”). Items are rated on a 4-point Likert-type scale (from 0 = “not at all” to 3 = “nearly every day*”*). Scores can range from 0 to 27; scores of 20 and higher are considered clinically significant. Internal reliability was acceptable in the current sample (T0: alpha = .75, T1: alpha = .76).

The General Anxiety Disorder (GAD-7; Spitzer et al. 2006) was used to measure anxiety symptoms. The GAD-7 is a 7-item scale assessing anxiety levels in the past 2 weeks (e.g. “feeling afraid as if something awful might happen”). Items are rated on a 4-point Likert-type scale (from 0 = “not at all” to 3 = “nearly every day”). Scores can range from 0 to 21; scores of 15 and higher are considered clinically significant. Internal reliability in the current sample was good (T0: alpha = .89, T1: alpha = .84).

Several questions were developed for this study relating to unpleasant experiences during the mindfulness course, perceived harm from the course, and support for any difficult experiences. The questions are shown in Supplementary Material S1. Some used Likert scales whereas others requested free-text responses. A written introduction to these questions noted that the practice of mindfulness can increase awareness of the full range of human experiences, including difficult thoughts, emotions, and sensations. Participants then were asked how often they had such experiences during the course (with response options ranging from “never” to “daily or almost daily”) and how upsetting these experiences were (response options ranging from “not at all” to “extremely”). A free-response question asked them to describe their unpleasant experiences during the mindfulness course in more detail. Next, harm was defined for participants as being “worse off, in any way, after the course, than you would have been if you hadn’t done the course”. Participants were asked how harmful the course had been for them (response options ranging from “not at all” to “extremely”) and a free-response question asked them to describe the harm in more detail. Finally, they were asked if they had sought support for any difficult experiences and, if so, from whom and how helpful the support was (see Supplementary Material S1 for further details).

#### Data Analyses

Means and standard deviations (*SD*s) were computed for symptom measures. Reliable change was calculated using the algorithm developed by Evans et al. (1998), who define reliable change as a function of the initial *SD* of the measure and its reliability. Using this algorithm, reliable change for the PHQ (depression) was a change in raw score of 4.81 or more in either direction; reliable change for the GAD-7 (anxiety) was a change in raw score of 3.96 or more in either direction. Using each measure’s cut-off points for clinical significance (described earlier), we further classified participants as showing “clinical change” if their score crossed the threshold for clinical significance in either direction. This yielded five categories of change: (a) “reliable and clinical improvement”, (b) “reliable improvement”, (c) “no reliable change”, (d) “reliable deterioration”, and (e) “reliable and clinical deterioration”.

Frequencies were computed for Likert ratings of unpleasant experiences and harm. Free-text responses about unpleasant experiences were examined to identify those falling into two categories previously mentioned that are familiar to experienced mindfulness teachers: (1) “difficult cognitions, emotions, or sensations” and (2) “negative thoughts and feelings about practicing mindfulness” (e.g. guilt or self-criticism about not practicing enough or feeling unable to do what the guidance suggested). Responses that did not fit neatly into these two categories were grouped into new emergent categories: (3) “recognition of personal habits or patterns”, (4) “descriptions of benefits along with difficulties”, and (5) “statements that the experience was not unpleasant accompanied by descriptions of experiences that had occurred”. Free-text responses about harm were not categorized because there were too few of them (most participants said they had experienced no harm). Instead, these responses were described and summarized. All free-text responses (anonymized) are provided in the Supplementary Materials S2–S7.

### Results

The mindfulness courses were well attended: 51% of participants attended all 8 sessions (*n* = 43), 32% attended 7 sessions (*n* = 27), 10% attended 6 sessions (*n* = 8), 6% attended 5 sessions (*n* = 5), and 1% attended 4 sessions (*n* = 1). Partial session attendance (i.e. the participant arrived late or left early from the session) was coded as attendance.

Mean levels of anxiety and depressive symptoms decreased from pre- to post-course (see Table 1). At baseline, four participants (5%) fell in the clinically significant range for anxiety. At post-course, this was reduced to one participant as the other three showed reliable and clinical improvement. An additional 20 participants (24%) showed reliable improvement; 56 participants (67%) showed no reliable change; and 5 participants (6%) showed reliable deterioration. No participants experienced reliable and clinical deterioration on anxiety. For depression symptoms, reliable and clinical improvement was not possible because no one had scored in the clinical range at baseline. Eleven participants (13%) showed reliable improvement; 71 participants (85%) showed no reliable change; and 2 participants (2%) showed reliable deterioration. No participant experienced reliable and clinical deterioration on depression.

**Table 1:** Change in symptoms and reliable improvement and deterioration in studies 1 and 2

	T0 M (SD)	T1 M (SD)	T2 M (SD)	Reliable and clinical improvement	Reliable improvement	No reliable change	Reliable deterioration	Reliable and clinical deterioration
Study 1	*n* = 84	n = 84	--					
Anxiety	4.86 (4.31)	3.32 (3.28)	--	4% (3)	24% (20)	67% (56)	6% (5)	0
Depression	5.10 (3.47)	3.40 (3.47)	--	0	13% (11)	85% (71)	2% (2)	0
Study 2	*n* = 74	*n* = 74	*n* = 60					
Global distress T1	12.14 (6.75)	9.11 (5.45)	--	14% (10)	5% (4)	77% (57)	0	4% (3)
Global distress T2	12.14 (6.75)	--	9.15 (5.61)	15% (9)	3% (2)	75% (45)	0	7% (4)

Participants’ ratings of unpleasant experiences and harm are shown in Table 2. Most participants (77%) reported that they “never” or “occasionally” had unpleasant experiences during the course; 22% had them more often. Of those who reported any unpleasant experiences (*n* = 56), most (88%) said that they found these experiences “not at all” or “somewhat” upsetting; 12% described them as “quite a bit” or “extremely” upsetting. Most (96%) described the course as “not at all” harmful; 3 (5%) participants described it as “somewhat” harmful. Seven participants reported that they sought support for difficult experiences: 5 from the mindfulness teacher, 1 from a family member or friend, and 1 from a doctor, counsellor, or mental health professional.

**Table 2:** Frequencies of unpleasant experiences and harm in studies 1 and 2

	Study 1 Post-course % (*n*)	Study 2 Post-course % (*n*)	Study 2 6-week follow-up % (*n*)
Frequency of unpleasant experiences associated with mindfulness practice during the course or follow-up period			
Never	33% (27)	34% (25)	27% (16)
Occasionally	45% (37)	27% (20)	35% (21)
Less than once/week	11% (9)	20% (15)	13% (8)
About once/week	10% (8)	9% (7)	15% (9)
Several times/week	1% (1)	5% (4)	3% (2)
Daily or almost daily	1% (1)	4% (3)	7% (4)
How upsetting were the unpleasant experiences? (for those reporting any unpleasant experiences)			
Not at all	43% (24)	33% (16)	19% (8)
Somewhat	45% (25)	59% (29)	66% (29)
Quite a bit	11% (6)	8% (4)	15% (7)
Extremely	2% (1)	0 (0)	0 (0)
How harmful was the course?			
Not at all	96% (80)	97% (72)	93% (56)
Somewhat	4% (3)	3% (2)	5% (3)
Quite a bit	0 (0)	0 (0)	2% (1)
Extremely	0 (0)	0 (0)	0 (0)

A free-response question asked participants to describe their unpleasant experiences in more detail. Responses were provided by 59 participants (71%). Examples (anonymized) are shown in Table 3; complete responses are in the Supplementary Material S2. Many described difficult emotions, cognitions, or sensations such as frustration, anxiety, distressing thoughts or memories, bodily pain or discomfort, and sleepiness. Several described patterns of thinking, feeling, or acting that they had observed in themselves as a result of practicing mindfulness. A few described self-criticism or negative emotions about not doing mindfulness practices correctly or not practicing enough. Several noted that they did not have unpleasant experiences but provided related observations and reflections (e.g. “not unpleasant but emotional in the first few sessions”) or described benefits along with difficulties.

**Table 3:** Examples of free-text responses about unpleasant experiences in study 1

Unpleasant emotions, cognitions, sensations	When exploring difficulties memories of my deceased friend were hard to deal with I had feelings of anxiety when focusing on my breathing. Agitation during formal practices. Had to stop a couple I would often fall asleep for very short moments during practices and feel very tired I experienced pain behind my eyes Feeling claustrophobic, fidgety, confronted with difficult thoughts Anxiety due to a past distressing personal experience
Negative thoughts/feelings about mindfulness practice	I got cross when I could not manage to “put my thoughts on a bench” in chapter 6 I also got frustrated if I could not focus on the practice as well as I wanted to. I also was very judgemental of myself when I did not do a practice right or forget to do the practice.
Personal patterns	It made me confront how my mind works which was that I think too much and find it increasingly difficult to switch off Recognising that I struggled to say I love myself. It was like [I] faced up to a sad reality Really made me focus on those areas that I do not like about my behaviour, particularly towards my family and those I love.
Not unpleasant but…	Not unpleasant but emotional in the first few sessions. It wasn’t unpleasant just normal anxiety maybe due to agitation of doing different tasks and needing to get them done.
Benefits along with difficulties	It made me realise that I was upset with my [family member having illness]…But the exercise made me realise that it was ok not to fix it, ok to feel. This felt liberating in the end. I had some feelings of inadequacy and guilt, which made me anxious/ possibly a little self-critical, but I do now feel empowered and very keen to work on and deal with these feelings

A separate free-response question asked participants to describe their experiences of harm in more detail. Although 81 participants responded to this question (see Supplementary Material S3), 73 of them stated that the course was not harmful. All eight of the remaining responses are shown in Table 4. Of these, three are from the participants who rated the course as “somewhat” harmful on the Likert item; the other five are from participants who rated the course as “not at all” harmful. Of the three who had rated the course as harmful, two seemed to qualify or contradict this rating: one noted that calling the experience harmful “may be a bit drastic” and the other stated that the difficulties were “in no way harmful or damaging.” The third mentioned managing pain and difficulty and finding time to practice.

**Table 4:** Free-text responses about harm in study 1

From the three participants who rated the course as somewhat harmful on the Likert item	Managing pain and difficulty as well as finding time for the meditations It made me think about some issues which I would have rather not considered which threw me for a while and caused some unpleasant memories and reactions - this impacted on my home life BUT this was only for a few days and with the practices that I was doing that week I managed to totally 100% deal with this, so to say it is “harmful” may be a bit drastic Initial feelings of worry/ guilt/ self-criticism when old emotions were unleashed, but I have already begun to accept and forgive these feelings and am hugely grateful for the mindfulness course as it has enabled me to recognise and deal with some of these thoughts and feelings. So on the whole, the very slight initial negative impact has been totally outweighed by the positive outcomes. The difficulties I had were in no way harmful or damaging, they were more like reality checks, making me aware of some old patterns of thinking/ acting that I no longer needed to follow.
From five participants who rated the course as not at all harmful on the Likert item	I did experience harm but I was upset after one of the sessions this was more to do with the timing. Described above [in the free-text question about unpleasant experiences] I did experience harm. I just stopped doing the exercises. It was a difficult time in our family and trying to tackle this jump in the mindfulness process at the same time was difficult and a little distressing.

Of the seven participants who experienced reliable deterioration in their anxiety or depression scores from T0 to T1, three reported that they “never” had unpleasant experiences during the course, three had them “occasionally”, and one had them “less than once a week but several times during the course”. These seven participants reported that they found these experiences “not at all” (*n* = 4) or “somewhat” (*n* = 3) upsetting. All seven participants described the course as “not at all” harmful. One participant sought support from a family member or friend for difficult experiences. The free-response comments for these seven participants were similar to those reported earlier. Examples include “Dealing with the emotions of [family member having illness] has been hard, but the course has helped me acknowledge them” and “Exploring difficulty brought back some unpleasant memories but my practice helped me to work through them”.

## Study 2

### Methods

#### Participants

Participants in study 2 were undergraduate and postgraduate students (aged ≥ 18 years) at the University of Oxford who signed up for one of the 8-week group-based mindfulness courses for students offered regularly by the Oxford Mindfulness Centre (OMC). Potential participants were provided with information about the course and contraindications to participation (such as serious mental or physical health concerns or recent bereavement) and were asked to make an informed choice about whether the course was suitable for them. Between October 2017 and April 2019, the online registration system asked each student who had signed up if they were interested in participating in a research study related to their experiences of the course. The research team emailed information to those who expressed interest and provided an opportunity to ask questions and consent to the study via an online form. During the study period, 296 students took part in ten mindfulness courses. Of these, 86 (29%) consented to participate in the study; 74 of them provided post-course data and 60 provided data at 6-week follow-up. Analyses described below are based on these final samples of 74 and 60, respectively.

The sample providing post-course data (*n* = 74) included 50 women, 23 men, and 1 who identified as other. Their mean age was 24 years (range: 18–49, *SD* = 5.37); 60% were postgraduate students and 40% were undergraduate students. The sample was 78% White British or White Other. Previous diagnosis with a mental health problem was reported by 34% (*n* = 25); of these, 16 reported that their difficulties were ongoing. Diagnoses included ADHD, depression, anxiety disorders, eating disorders, post-traumatic stress disorder, and depersonalization-derealization disorder. A lifetime history of potentially traumatic experiences, broadly defined to include death of close friends or family members or parental divorce, was reported by 74% of participants (*n* = 55). For 53% of the sample (*n* = 39), scores on the CORE-10 fell within the healthy or low-level range; the remaining 35 participants had scores indicating mild (24%), moderate (14%), moderate-severe (4%), or severe distress (5%).

#### Procedure

Self-report data were collected at three time points: the week prior to the first session (T0), the week following the end of the 8-week course (T1), and 6 weeks later (T2). Participants were reimbursed £20 if they completed the questionnaires at both of the first two time points and an additional £10 for T2 (a maximum of £30 in total). All communication between the research team and participants was via email. Consent forms and self-report questionnaires were administered online using Qualtrics software. Participant safety was addressed with the study’s risk and safeguarding protocol which included providing information about sources of support to those reporting significant distress, contacting those reporting suicidal ideation for further assessment, and when appropriate, alerting the college nurse. The University of Oxford Research Ethics Committee approved the study (16/10/2018; ref. R52786/RE004).

##### Mindfulness Program

As in study 1, the mindfulness program was “Mindfulness: Finding Peace in a Frantic World” (M-FP; Williams and Penman 2011), an adaptation of MBCT with 8 weekly 90-min sessions designed for nonclinical populations. Each group had up to 30 participants (*M* = 29.6, *SD* = .97) and was taught by a qualified mindfulness teacher from the OMC who met the good practice guidelines described in study 1. The course fee was £65. The mindfulness teachers were not involved in the research study and were unaware of which students were study participants.

#### Measures

For a larger study of outcomes of the mindfulness course, participants completed measures of global distress, wellbeing, resilience, mindfulness, and self-compassion. These outcomes are reported in a separate paper (Medlicott et al. under review). For the present study, we discuss only the measures of global distress, unpleasant experiences and harm. Participants were asked to provide their age, gender, ethnic group, education, mental health history, and history of trauma.

The Clinical Outcomes in Routine Evaluation (CORE-10; Connell and Barkham 2007) was used to measure psychological distress. The CORE-10 consists of 10 statements about thoughts and feelings (e.g. “I have felt tense, anxious, or nervous”) and asks respondents to rate how often they felt this way over the past week using a five-point scale ranging from 0 (“not at all”) to 4 (“most or all of the time”). A total distress score is calculated by summing the items. Scores can range from 0 to 40; a score of 11 or higher is considered clinically significant. The CORE-10 has shown good validity, internal consistency, and sensitivity to change (Connell and Barkham 2007). Internal consistencies in the current sample were good (T0: alpha = .84, T1: alpha = .81, T2: alpha = .82).

The same set of questions described in study 1 (Supplementary Material 1) was administered to assess unpleasant experiences and harm.

#### Data Analyses

Data were analyzed using the same methods described in study 1.

## Results

Of the 74 who provided T1 data, 49% attended all eight sessions (*n* = 36), 22% attended seven sessions (*n* = 16), 15% attended six sessions (*n* = 11), 9% attended five (*n* = 7), and 5% attended 4 sessions or less (*n* = 4).

At baseline, 45 participants (52%) met the clinical cut-off for global distress on the CORE-10; this decreased to 31 (42%) post-course and to 17 (27%) at 6-week follow-up. Mean levels of global distress decreased from pre- to post-course and the decrease was maintained at 6-week follow-up (see Table 1). Reliable change on the CORE-10, computed using the same methods as in study 1, was a change of 7.48 or more raw score points in either direction. From pre- to post-course, 10 participants (14%) showed reliable and clinical improvement; 4 (5%) showed reliable improvement; 57 (77%) presented no reliable change; and 3 (4%) showed reliable and clinical deterioration. At follow-up, 9 participants (15%) showed reliable and clinical improvement; 2 (3%) showed reliable improvement; 45 (75%) presented no reliable change; and 4 (7%) showed reliable and clinical deterioration.

Responses to the Likert-scale questions about unpleasant experiences and harm are shown in Table 2. At T1 (post-course), 61% reported having unpleasant experiences “never” or “occasionally”; the remainder had them more frequently. Of those who reported unpleasant experiences (*n* = 49), 92% described them as “not at all” or “somewhat” upsetting. Most (97%) reported being unharmed; two reported being “somewhat” harmed. Nine sought support for difficult experiences from the mindfulness instructor, a family member, friend, or healthcare professional.

At T2 (6-week follow-up, *n* = 60), 73% of participants reported having unpleasant experiences associated with mindfulness practice at least “occasionally” during the 6-week follow-up period. Most (85%) described these experiences as “not at all” or “somewhat” upsetting. Ten sought support, most from a family member, friend, or healthcare professional. Fifty-six (93%) participants reported being “not at all” harmed by the mindfulness course, 3 (5%) reported being “somewhat” harmed, and 1 reported being “quite a bit” harmed.

Examples of free-text responses about unpleasant experiences are shown in Table 5 (complete data are in the Supplementary Materials S4 and S5). Responses at T1 and T2 were similar and are combined. As in study 1, many of these involved difficult thoughts, emotions, and physical sensations; others described recognition of personal patterns. Several described self-criticism about not doing the meditation practices correctly, or not practicing enough, and associated feelings of guilt, frustration, agitation, and self-directed annoyance. A few noted that their experience was not unpleasant but provided related observations; others described benefits along with the difficulties.

**Table 5:** Examples of free-text responses about unpleasant experiences in study 2

Unpleasant emotions, cognitions, sensations	Anxiety and fatigue Felt a bit overwhelming at times Stress related to memories that arose Sleepiness very often. Especially with certain practices that I found more difficult (like exploring difficult thoughts) If I was already in pain then focusing on it would make the sensation more intense. Tension in shoulders/neck Panic attacks and difficulty breathing
Negative thoughts/feelings about mindfulness practice	When I noticed my posture wasn’t perfect I was annoyed with myself…that it was my fault, that I wasn’t doing the practice properly There was sometimes guilt if I did not do the practice one day Sometimes irritation at myself for not doing mindfulness, feelings of having failed or not being good enough. These latter feelings if I do not feel I’ve sustained concentration/mindfulness throughout a meditation.
Personal patterns	I have come to realise that I have very low self-confidence and do not think that I can be loved by others, which has been quite painful…I also realised that I do not let myself feel much when I judge that my emotions are “silly” or irrational Whilst doing the difficulty meditation, I sometimes found myself experiencing quite strong emotions, which I had not realised existed. The “Wishing yourself well” meditation was also quite painful - I found it (and still do) find it very difficult to wish myself well. It has been very enlightening, however, to realise that I had these thought patterns and behaviours…
Not unpleasant but…	Sleepiness - I would not necessarily describe it as unpleasant, but it did interfere with the completion of the practice on several occasions, especially during the body scan meditation.
Benefits along with difficulties	I became aware of some back pain that my body had previously attenuated. It’s actually quite painful. As I have become more mindful of the pain, I made adjustments to how I sleep and my posture at other times. I still have some pain, but on the whole it seems to be improving. Spending more time thinking about my state of mind and mental health led me to seriously question some aspects of my life and this led to distress and confusion, but overall this resulted in changes that have been really beneficial.

All six free-text responses about harm are shown in Table 6 (see also Supplementary Materials S6 and S7). These described increased stress levels, difficult thoughts, and awareness of distress and unhappiness. Two mentioned potential overall positive impact, one noted that the harm was not long-term, and the other three described only negative outcomes.

**Table 6:** Free-text responses about harm in study 2

Post-course	I feel like the money I spent on it was wasted, as I did not attend the whole course and it created extra stress for me when I avoided going It made me stress a bit more, but again, may have been due to external reasons during term. Occasionally it was helpful, but I’m not sure whether overall it has made a positive impact or not
6-week follow-up	Having to deal with the “buried” issues which surfaced during the course…actually increased my stress levels, and resulted in me having to go home for a few days to unwind. However, after starting to deal with some of these issues, I think it will be overall positive. I’ve had thoughts that it’s better not to have to pay attention to, paying them attention at the present time would be debilitating and make it hard to function in everyday life expectation of a cure which simply wasn’t met, − attempting to dismiss my thoughts mindfully often draws me further into them than simply ignoring them… Feeling like an over-rational robot… Spending a lot of time trying to work out how to escape from society whilst also feeling lonely, which admittedly I did do before but less so… the slightly and moderately distressing thoughts are easy to dismiss which has left me with only the big ones. “somewhat” is probably an exaggeration - throughout the course I was still in shock regarding [a recent bereavement] (though I did not realise that at the time) and it was too soon for me to do the course … at some points it made me aware of how unhappy/distressed I still was … so in that way it was harmful, although there has been no long term harm whatsoever

The three participants who showed reliable and clinical deterioration at post-course reported unpleasant experiences related to the mindfulness course ranging from “occasionally” to “about once a week”. All three described the unpleasant experiences as “somewhat” upsetting. Free-response descriptions of unpleasant experiences included “Bringing up an old relationship hurt for a while”, *“*I became slightly obsessed with these thoughts for a week or so, but the meditations helped me approach the issues and, in a way, resolve them”, and “when I noticed my posture wasn’t perfect I was annoyed with myself”. All three of these participants rated the course as “not at all” harmful.

At 6-week follow-up, four participants showed reliable and clinical deterioration. Of these, two had shown this deterioration at post-course whereas two had not. Three of these four participants reported that they had unpleasant experiences “occasionally”, one had them “daily or almost daily”. Two rated these unpleasant experiences as “not at all” upsetting, two as “somewhat” upsetting. Free responses included, “Sometimes irritation at myself for not doing mindfulness, feelings of having failed or not being good enough” and “I notice that my thoughts are unpleasant”. One of these participants reported that course had been “quite a bit” harmful and wrote that it brought awareness to distressing thoughts and thought patterns that were difficult to deal with and felt overwhelming. This participant also wrote, “I answer that mindfulness has been quite a bit harmful in the next question which is true but it has helped in other ways”.

## Discussion

Prevention of harm is a primary ethical duty in the Hippocratic tradition and requires knowledge of harms that might occur. Research on MBPs has provided little information about the likelihood of harm to participants. A recent review (Baer et al. 2019) proposed a framework for research and recommended increased monitoring of both expected and unexpected unpleasant experiences and harm in evidence-based MBPs. The aim of this study was to explore the frequency and severity of unpleasant experiences and harm in participants in an 8-week adaptation of MBCT for the general population. Data were collected from two samples: schoolteachers completing a personal mindfulness course before teaching mindfulness to their pupils, and university students completing a mindfulness course to support their wellbeing. Participants completed self-report measures about their levels of symptoms and responded to questions about unpleasant experiences and harm.

For both samples, about two-thirds of participants reported unpleasant experiences during the course. Generally, these were consistent with the types of difficult experiences that mindfulness teachers expect (Segal et al. 2013), including unpleasant emotions, thoughts, and sensations, and negative thoughts and feelings about practicing mindfulness. Such experiences are expected because establishing a regular practice is difficult and because mindfulness practice cultivates increased awareness of whatever is happening in the present, whether it is pleasant, unpleasant, or neutral. Goals of the mindfulness program used in these studies include increased awareness and appreciation of pleasant experiences as well as learning to work skillfully with the difficult emotions, cognitions, and physical discomforts that are inevitably part of life. Participants are informed that awareness of the full range of human experience can be challenging. Most participants who reported unpleasant experiences rated them as not at all or only somewhat upsetting, suggesting that they found these difficulties to be manageable. Some of the free-text responses noted that these difficult experiences during the course led to important benefits.

The proportion of participants who reported that they had been harmed by the mindfulness course ranged from 3 (student sample at post-course) to 7% (student sample at 6-week follow-up). For the teacher sample, 4% reported harm (post-course). These percentages are consistent with the findings for deterioration on the symptom questionnaires: 2% for depression and 6% for anxiety in study 1 (reliable but not clinical deterioration), and 4–7% for global distress in study 2 (reliable and clinical deterioration). These percentages are also similar to those reported in the psychotherapy literature, which has consistently found that between 3 and 10% of clients show deterioration in symptoms or functioning (Lambert 2013), and in the meditation literature, where Baer et al. (2019) found that adverse events were reported in 0–10.6% of participants.

Reliable deterioration on symptom questionnaires is not necessarily attributable to the mindfulness program but could have occurred for other reasons. Indeed, there was very little overlap between those showing reliable deterioration on questionnaire measures and those describing the mindfulness course as harmful. In study 1 and study 2 at post-course, all participants who showed reliable deterioration in questionnaire scores described the mindfulness program as not at all harmful, suggesting that they did not attribute their increase in symptoms to the mindfulness program, or did not perceive their symptom increases as harmful. Conversely, all participants in study 1 and study 2 at post-course who said they had been harmed by the program showed no reliable deterioration in their symptoms. There was only one exception to this pattern: a participant in study 2 at 6-week follow-up who reported harm from the program and also showed reliable deterioration in mental health.

Whilst harmful experiences were rare, these findings highlight the need for mindfulness teachers to manage expectations about both benefits and difficulties that may occur in a mindfulness-based program. Before the program begins, participants should be informed that the practice of mindfulness can be challenging and uncomfortable, that the course will teach skills for working with difficulties, and that opportunities to practice the skills with difficult experiences that arise in meditation can be valuable learning experiences. Assessment and orientation procedures should be carefully designed to provide this information and assess whether this is a good time for prospective participants to complete the course. For those who have already begun a course before difficulties arise, discontinuation may be advisable, possibly with referral to other services. Mindfulness teachers need to be alert to and aware of these issues so that they can support participants as appropriate. Our findings also suggest that self-criticism and negative emotions about mindfulness practice occur despite the fact that teachers attempt to work constructively with difficulties in finding time to practice and often say that there are no right or wrong experiences during mindfulness meditation. Increased awareness and open supportive discussion of the common tendency toward self-criticism may be necessary.

### Limitations and Future Research

This study has several important limitations. First, although both samples included some participants with significant mental health symptoms, the study used general population samples and findings may not generalize to clinical populations. General population samples are important because MBPs are increasingly used in nonclinical settings such as schools and workplaces. Second, whilst reliable deterioration in our samples was similar to rates reported in the psychotherapy literature and the meditation literature, the absence of control groups means that we could not compare our rates of reliable deterioration to the rates that would be seen in a group receiving no training or an alternative program. This is important because the risks of participating in any program must be balanced against the risks of doing nothing or doing something else. Third, the study was not designed to investigate sources of harm. Baer et al. (2019) reviewed the literature on harm in MBPs and suggested three potential sources of harm which are likely to be interacting: program factors, participant factors, and teacher factors. Additional study of these potential sources of harm is needed, especially as the teachers in this study were well trained. Fourth, the questions about unpleasant experiences and harm were added to two larger studies, each designed for its own purposes (described elsewhere) and using different outcome measures. Accordingly, the relationships between unpleasant or harmful experiences and symptom change are not directly comparable across the two studies. Finally, we used ordinary language (unpleasant, harm) rather than technical terms (adverse events, serious adverse events) because we were interested in participants’ experiences, in their own words and from their own perspectives, and wanted to facilitate their ability to describe them. Future work should continue to clarify how such experiences can best be understood in the context of skills training and psychological change, which can be difficult and challenging without causing harm.

In future studies, it may be helpful to ask more explicitly about the balance of benefits and harms or pleasant and unpleasant experiences. The questions we developed for this study asked only about unpleasant and harmful experiences and included no questions about pleasant experiences or benefits. Even so, several participants volunteered information about benefits arising from the unpleasant experiences. As noted earlier, a few participants who rated the course as harmful on the Likert item contradicted themselves in the free-text responses, suggesting that their understanding of “harm” may not have been clear. Studies that include interviews of participants reporting harm might facilitate greater clarity about participants’ perceptions of harm and how these correspond to widely cited definitions of harm (Dimidjian and Hollon 2010; Duggan et al. 2014). Interview methods would also allow more systematic study of the types of difficult experiences common in mindfulness programs. If the preliminary categories identified here are replicated in a broader range of samples, they could provide evidence-based guidance for mindfulness teachers about how to discuss the likely risks and benefits of mindfulness courses with their potential participants.

In summary, our findings add to the literature on unpleasant and harmful effects of MBPs by asking participants in two samples about their experiences with such effects using open-ended opportunities to describe them in combination with Likert scales. Whilst reports of harm were rare, challenging and unpleasant experiences were common, and it seems clear that teachers and participants must understand that such experiences are likely and learn to work skillfully with them. Moreover, the minimal overlap between participants who reported that the MBP was harmful and those showing reliable deterioration on symptom questionnaires suggest that the experience of harm is not necessarily captured by outcome questionnaires and should be explicitly assessed in other ways. Finally, our findings suggest that more detailed study of unpleasant experiences and harm could increase understanding of these experiences in the broader literature on psychotherapeutic interventions and other skills training programs.

## Supplementary Information

ESM 1 (DOCX 50 kb)

## Data Availability

Following the International Committee of Medical Journal Editors (ICMJE), all of the individual anonymized and completely de-identified participant data are available for any analytical purpose that is related to achieve aims in the present study upon reasonable request to researchers (a) who provide a methodologically sound proposal and (b) whose proposed use of the data has been approved by an independent ethical review committee. The database will be provided by the corresponding author to interested researchers that meet the aforementioned criteria.
