# Supplementary material for: Frequency of Self-reported Unpleasant Events and Harm in a Mindfulness-Based Program in Two General Population Samples
Source: Mindfulness (N Y). 2020 Dec 2;12(3):763–74. doi: 10.1007/s12671-020-01547-8 (PMC7920887; doi:10.1007/s12671-020-01547-8)
Supplement: Supplementary file 1 — (DOCX 50 kb) [file 12671_2020_1547_MOESM1_ESM.docx]

**SUPPLEMENTARY MATERIALS**

Frequency of self-reported unpleasant events and harm in a mindfulness-based program in two general population samples

**Supplementary Material S1:** Questions about unpleasant experiences and harm

**There has been a lot of research into the benefits of mindfulness courses but much less on any negative experiences people may have. These questions ask about unpleasant or negative effects that you may have experienced.**

*The practice of mindfulness involves becoming aware of the full range of our experience: pleasant, unpleasant, and neutral. This can be challenging, as it sometimes puts us in touch with difficult thoughts and feelings. Learning to manage these thoughts and feelings is part of the mindfulness course and takes time. While it is going on, people taking part in a mindfulness course can experience unpleasant thoughts and feelings such as agitation, sleepiness, uncertainty, negative emotions, or other discomforts in the mind or body.*

How often did the mindfulness course lead to you having unpleasant thoughts or feelings?

- Never
- Occasionally
- Less than once a week, but several times during the course
- About once a week
- Several times a week
- Daily or almost daily

How upsetting were these experiences?

- Not at all
- Somewhat
- Quite a bit
- Extremely

Can you describe the unpleasant experiences you had during the mindfulness course in a more detail?

**Harm**

The next questions ask about whether you think that doing the mindfulness course caused you any harm. **By harm, we mean: were you *worse off in any way, after the course, than you would have been if you hadn’t done the course*?**

How harmful was the course for you?

- Not at all
- Somewhat
- Quite a bit
- Extremely

Can you describe the harm you experienced during the course in a more detail?

***If you did not experience unpleasant effects or harm, then please stop here, and thank you for your time.***

***If you experienced any negative or unpleasant effects or harm during the course, please could you answer the following questions***

Did you tell anyone about the unpleasantness or harm, or seek any help or support?

- I had difficulties, but I did not seek any help or support
- I had difficulties and I did tell someone / seek support

*If you didn’t tell anyone:*

Can you describe why you did not seek any help or support for your difficulties?

*If you did tell someone:*

Who did you approach?

- The mindfulness instructor
- A family member or friend
- A doctor, counsellor or other mental health professional
- Someone else (support groups, online resources) [please describe]

Did you receive adequate support in managing the difficulties you were experiencing? Please choose the number that best describes your experience:

- 1 (no support or unhelpful support)
- 2
- 3 (moderately helpful support)
- 4
- 5 (very helpful support)

If you’d like to add any comments about the support you received, please do so here:

**Thank you. Your responses to these questions will be a great help in understanding the complete range of effects of mindfulness courses.**

**Supplementary Material S2:** Participant reports of unpleasant experiences in Study 1

| It made me confront how my mind works which was that I think too much and fine it increasingly difficult to switch off. This was a surprise to me, as although I am aware my mind is whirring, trying to control it was quite difficult. It did make me reassess whether I ever truly appreciate the here now as my mind is always inclined to move on to the next thing. |
| --- |
| I didn't have any 'unpleasant' experiences. At times I got a little sad but felt that the group and of course our teacher and the practice helped me get over these feelings very quickly. |
| I think the focus on self perhaps led to more self-awareness and therefore more self-scrutiny, and perhaps criticism of self arising from that scrutiny. |
| At some points in the course I tried to address a very difficult issue that had happened and found that I focussed all of the time on this issue. I think that I chose too big an issue to start with. Once I realised this I was fine. |
| Sometimes the longer meditations were difficult because they felt repetitive. I got frustrated because when I started the course I found them liberating but when I listened to a meditation on the CD more than a couple of times I found it difficult to be truly mindful and present, I found my mind was trying to do the recorded meditation on autopilot constantly! It took far greater effort to constantly break that. I found it easier with a real life instructor present. Also during the exams period I felt I needed more urgency and a bit of 'stress' in order to perform my job properly. I found that mindfulness made me feel less urgency and stress about tasks that needed to be done quickly and at short notice, which meant I didn't get some things done. I know this sounds the opposite to what mindfulness is meant to do (and generally mindfulness overwhelmingly does let me perform better in my job and life). But aspects of my job are so demanding at certain points in the year you need a real 'fight' response to get through some of it, as the workload is more than I am given enough time or pay to do. Mindfulness makes me put myself first - and actually, sometimes, I had to put the work first, rightly or wrongly, otherwise it wouldn't have got done. As a result I did less mindfulness in that period and then picked back up on it after I'd got the students through the last push of coursework and exams. Also I found the facing difficulty meditation extremely hard, so I avoided doing it and only did it twice. However after I had done it, in the following days and weeks I found the very issue I had made the focus of 'facing difficulty' gained momentum in a positive direction. I this was an issue that has plagued me for years. |
| Dealing with the emotions of [family member having illness]…has been hard, but the course has helped me acknowledge them. |
| Befriending wasn't really upsetting, but made me look at an unpleasant situation. This was ok |
| Just thoughts of things that might happen to me and my family, for example death |
| It was during the face difficulties week, it made me realise that I was upset with my [family member having illness] and knowing there was perhaps no solution; more than I had realised. At 1st I was saying it was silly as he wasn't dying. But the exercise made me realise that it was ok not to fix it, ok to feel. This felt liberating in the end. I also realised that I did have inadequacies feelings which was unsettling but again as I understood them through the practices I became fine very quickly. |
| I [got some bad news about] my job during the course … this meant I had to focus on how I was feeling which I found incredibly difficult and led to a few sleepless nights. It also meant I dreaded having to complete home practises for fear of having the same feelings each time I completed them. |
| On one occasions while doing a practice got quite overwhelmed by feelings of having too much to do, being so busy - quite unpleasant |
| I found some of the being kind to yourself exercises difficult |
| I got cross when I couldn't manage to 'put my thoughts on a bench' in chapter 6. I found that I couldn't take this step for a long time. I think partly I was trying to tackle a problem that was to large, and I needed to practice on smaller problems, but by thinking about them and not being able to control it I became more emotional about them, this affected my sleep as I was worrying. However I also suspect that I had to go through this to learn how to achieve this skill which I have now managed |
| I wouldn't say unpleasant. At times the meditations made me sleepy because I was already tired. I did not warm to the difficulty meditation probably because it seemed paltry to think of a difficulty when I was dealing with bereavement. On the other hand the befriending one was excellent for me. |
| anxiety when completing the difficulties mindfulness week - my mind finds it hard to concentrate on small difficulties and kept wandering to the big ones and the emotions of these would then flood back. |
| References to the breath as an anchor brings up painful thoughts about a [bereavement] |
| Bringing attention to difficulty - I dislike flying and was due to fly that week. When I thought about it and recognised my feelings, both physical and emotional, it actually made me feel more anxious. |
| I didn't have unpleasant experiences. It was interesting observing my bodies reaction to unpleasant thoughts though |
| Didn't have any - some mild irritation with some of the practices and the wording of the book got right on my nerves at first but nothing complex or protracted. |
| Emotions which had been shelved were strong in their insistence when resurfacing at times and there were real frustrations with managing the 'freight train' of emotions when first loosened into conscious thought. I needed to learn not to fall into the default position of talking about them and allow then to simply be there: that was hard. |
| Feelings from my past. |
| I often over think situations. I had a retrospective sense of guilt to situations that I know I had previously over reacted to. This also recurred on occasions when I had not made time for mindfulness practice and when subsequently I allowed life situations to get to me. |
| Some more disturbing emotions were brought to the surface in one particularly difficult week but I was able to work thorough these calmly and clearly with the mindfulness teacher and to view them with some equanimity. |
| My experiences weren't really unpleasant. They came to mind but I felt able to let them go and not dwell too much on them. |
| One the practice where you had to think about your worries; I felt as though I had resolved the difficulty that kept coming into my head and I thought I had put it behind me. It was upsetting to have it brought back to the foreground of my mind again. |
| Not unpleasant but emotional in the first few sessions. |
| Greater self awareness around a particular unpleasant event when I was a teenager. |
| Sometimes the course caused me to think about some unpleasant experiences from my past. I also got frustrated if I couldn't focus on the practice as well as I wanted to. |
| Weeks 1-4 were really nice, very calming, I had no adverse effects. I have always been quite aware of how my emotions, moods, thoughts are interrelated, so found the early weeks enlightening and useful. But during weeks 5-7, it brought up emotions I had been suppressing, especially the Exploring difficulties meditation. And the Befriending meditation made me more aware of how little I was taking care of myself. I felt quite vulnerable and alone. So I had some feelings of inadequacy and guilt, which made me anxious/ possibly a little self-critical, but I do now feel empowered and very keen to work on and deal with these feelings myself through meditation this summer. |
| In the past I have tried to avoid unpleasant experiences so sitting with difficulty was hard |
| When exploring difficulties memories of my deceased friend being hard to deal with |
| Exploring difficulty brought back some unpleasant memories but my practice helped me to work through them |
| Brought on anxiety due to a past distressing personal experience. |
| unpleasant memories, awareness of the effect these events still have on my, the body, impulse and emotion sensations that accompany the memory etc but using the 'workbench' and different tools I have actually found I am more able to deal with these memories now. also on-going impulses to do things can be avoided by recognising what is happening |
| Mainly just working with difficulties made me consider difficult situations from the past, but also helped me to rationalise how I think about them. |
| I had feelings of anxiety when focusing on my breathing. I felt short of breath when I had to concentrate on my breathing. |
| Just feeling anxious or restless during some of the home practice. |
| Agitation and frustration. |
| During the difficulties session when discussing/using the workbench for problems I experienced pain.. In the group we discussed strategies that could help |
| frustration walking up and down |
| Sleepiness, an unwillingness to focus on difficulty but have learned to cope better with this. |
| when I was doing the mindful movements track I often felt very tearful after. |
| It was only during the turning towards difficulty meditations. Felt anxious but was interested in how my body reacted. |
| Agitation during formal practices. Had to stop a couple |
| During the body scan I was having a series of tests done in hospital. It made me very aware of how I was feeling |
| I would often fall asleep for very short moments during practices and feel very tired. I struggled at times to focus and concentrated on staying awake. This was uncomfortable and made me reflect on the amount of sleep I was getting to what my body and mind needed. I also was very judgemental of myself when I did not do a practice right or forget to do the practice. I learnt eventually not to be judgemental and accept what had happened. I sometimes felt agitated and frustrated, but this was more week 3-4 - which was when I wanted to give up. After that I enjoyed the practice sessions rather than getting inpatient and frustrated with them. |
| At the beginning I felt agitated at points as I was getting used to the practice. At points I suffered from sleepiness, particularly when doing the body scan. I had to adjust the way I sat during these sessions to ensure I was fully aware. The first time we practiced the exploring difficulty meditation I found it extremely difficult and overwhelming. I was very subdued for the rest of the session and avoided practicing it again for several days. When I eventually tried again it was much easier and less emotional. I believe the negative experience I had was because I was feeling emotional that day due to external factors and so it emphasised those feelings. After that day those feelings became less and so the practice became easier. |
| They bought to my attention things that I hadn't been thinking about - sometimes I felt more stressed or anxious when I was in the sessions, than I did before I had started. |
| It's wasn't unpleasant just normal anxiety maybe due to agitation of doing different tasks and needing to get them done. |
| Feeling claustrophobic, fidgety, confronted with difficult thoughts |
| I couldn't manage the mindful movement activities because I have a disability and I have developed a number of coping strategies to manage and ignore the pain; mindful movement made this impossible and I was concerned that I wouldn't be able to maintain my control of these feelings. |
| Being mindful made me aware of how unhealthy I am - physically. It also made me aware of how horrible I am to myself and how much self loathing I feel. I also want to seek further counselling now as I realise I am holding onto painful emotional issues that are affecting my present. |
| I suffer with health anxiety and sometimes focussing on feelings and sensations in my body caused some anxiety and led to negative thoughts about pains and twinges being the result of an illness. If the recording suggested I may feel agitation, or pain in my body I usually did and this was uncomfortable and caused agitation. |
| Recognising that I struggled to say I love myself. It was like faced up to a sad reality and realised how can I expect someone else to love me if I can’t love myself. |
| Exploratory rather than discomforting |
| The main example I can think of is when we wrote a letter to ourselves in the future, giving ourselves advice on how we should be more mindful… That activity really made me focus on those areas that I do not like about my behaviour, particularly towards my family and those I love. This was difficult to do, but rewarding in the sense that I realise what I can work on and now have the ability to accept that I'm not perfect and that I just need to try to work on it more. |
| I felt the act of kindness was selfish and had to come to terms with why it was being done- I thought about it and decided it was about having better feelings and NOT about me feeling better. This allowed me to complete the practice more easily. I was uncomfortable with the Exploring the Difficulty because I like to: rationalise, analyse and sort out how to solve a problem with a view to moving forward. This was a big challenge for me. |
| Dealing with difficult thoughts caused some sleep disturbance but I felt it help me understand them in the long run |
| Just becoming aware of how I had given little thought to the feelings of others. |

**Supplementary Material S3:** Participant reports of harm in Study 1

| no |
| --- |
| I didn't experience any harm |
| None to report |
| no harm experienced |
| No |
| I didn't feel harm, only the unpleasant anxious sensations when concentrating on my breathing |
| I did not feel that the course had harmed me, it has given me the insight to face myself in more depth. |
| It didn't |
| No harm at all |
| No harm |
| No harm |
| I had no harmful experiences |
| none |
| No harm experienced. |
| None |
| none |
| None. |
| Didn't feel any harm |
| I did experience harm but I was upset after one of the sessions this was more to do with the timing. |
| No harm at all |
| I didn't experience anything that I felt was harmful |
| described above |
| I did not experience any harm. |
| None |
| No harm |
| None |
| As described earlier- initial feelings of worry/ guilt/ self-criticism when old emotions were unleashed, but I have already begun to accept and forgive these feelings and am hugely grateful for the mindfulness course as it has enabled me to recognise and deal with some of these thoughts and feelings. So on the whole, the very slight initial negative impact has been totally outweighed by the positive outcomes The difficulties I had were in no way harmful or damaging, they were more like reality checks, making me aware of some old patterns of thinking/ acting that I no longer needed to follow. I discussed these with our mindfulness instructor during the meetings, so did not need to seek additional support |
| none |
| none |
| I did experience harm |
| N/A |
| N/A |
| N/A |
| Didn't have any |
| none |
| I just stopped doing the exercises |
| n/a |
| None |
| I did not feel any harm. The course allowed me to learn how to deal better with stress and know that I had more time than I thought I did. |
| None |
| NA |
| N/A |
| none |
| It was not harmful |
| I do not feel the course caused me harm. It was overall a very positive experience. |
| na |
| None |
| I was much better off for doing this course. |
| I didn't experience harm. |
| N/A |
| NA |
| none |
| It was a difficult time in our family and trying to tackle this jump in the mindfulness process at the same time was difficult and a little distressing. |
| I don't think the course was harmful as such; rather, it made me look at how my mind works and while that might have highlighted some areas for improvement, it wasn't harmful to learn that about myself. |
| None |
| I think the course overall was beneficial, even if it did lead to a greater sense of self-awareness which had both positive and negative consequences. |
| NA |
| N/A |
| Absolutely none. It was a very pleasurable experience |
| I didn't |
| It wasn't really harmful |
| it made me think about some issues which I would have rather not considered which threw me for a while and caused some unpleasant memories and reactions - this impacted on my home life BUT this was only for a few days and with the practices that I was doing that week I managed to totally 100% deal with this, so to say it is 'harmful' may be a bit drastic |
| No harm Just was really aware of how stressed I was during one practice it became quite overwhelming - not harmful Made me aware of how stressed I was feeling |
| managing pain and difficulty as well as finding time for the meditations |
| It wasn't harmful |
| . |
| N/A |
| Did not experience |
| No harm whatsoever. |
| I didn't experience any harm. I felt it was a positive experience although some weeks were more interesting and poignant than others. |
| No harm |
| none |
| na |
| none |
| none |
| NA |
| N/A |
| na |
| not harmful |
| None |
| Nil. |
| It hasn't harmed me but it changes aspects of who I am when I am doing mindfulness regularly. For example it made me more relaxed about feeling COMPLETELY responsible for the students’ progress. It gave me a different attitude about it. I didn't want to do anything that I felt was rushed or to the detriment of my wellbeing. So I worked at a pace that was as efficient and as healthy as possible - as a result some things not a lot, but some didn't get done. However [some of my other work] improved outside of school. I also felt that I wanted to make the students more responsible for their actions or lack of work and I wanted to help them do this and develop a more independent work ethic. This in itself is a positive thing but some students didn't manage to rise to the challenge… |

**Supplementary Material S4:** Participant reports of unpleasant experiences in Study 2, Time 1

| A big reason I took part in the course was to combat intrusive thoughts, and early on some of the meditations brought me face to face with these thoughts. I was however able to deal with them by refocusing on breathing etc. |
| --- |
| Anxiety and fatigue, but I would have felt these emotions/sensations whether or not I had done the mindfulness course. |
| As previously mentioned, I completely fell asleep during the first in-class body scan. I did find the body scan caused me to fall asleep. Similarly, the sitting practices did promote a feeling of sleepiness, rather than alertness. I am hoping with more practice that I can train my body to be alert rather than asleep while meditating, but this did prevent me from being motivated to do the practices at home during the day. I did sometimes become agitated during practices as I have a back problem and I find it difficult to sit still without physically adjusting my position frequently in order to remedy my back by manually cracking it (though this is not medically advised) |
| At the very beginning of the course it was especially the inability to breathe properly. My breath was really short and shallow. It made me very agitated and even more stressed. I felt I couldn't take a proper breath and didn't understand what was going on and why. I had problems with sleeplessness and especially at the beginning of the course I felt like my eyes were still racing when I was meditating and that was keeping me awake. My eyes were tired, but too agitated in a certain sense to calm down and "let" me go to sleep. Sometimes I got annoyed with myself because of my posture. In the first six weeks I was quite obsessed with my proper posture - sitting upright, with my chin tucked, etc. - and I was checking it too much during the meditations, not allowing myself to be fully immersed in them. Also, when I noticed my posture wasn't perfect I was annoyed with myself. I felt like I was trying hard enough, that it was my fault, that I wasn't doing the practice properly and would not get out of it what I'm supposed to. I then discussed it with my teacher and he said I should allow my body to be in a position it wants to be in and then I relaxed more about it. Often when I closed my eyes to meditate, especially in the standing position, in my mind's eye my body was distorted. If I opened my eyes and checked I wasn't at all as distorted as I thought. But then when I closed them again I still mentally saw myself crooked - hunched posture, uneven shoulders and legs, my body skewed and twisted. |
| Awareness of : discomfort in my body, fatigue |
| Bringing up an old relationship hurt for a while, as I thought thinking about my ex was to be avoided. My ex had recommended the course, so I associated mindfulness with her, and therefore for a while the sessions turned into just thinking about her and feeling that I wasn't doing the course properly. Week 6 really really helped with this. The approaching difficulty meditation brought up several things that I have always worried about/disliked myself for. I became slightly obsessed with these thoughts for a week or so, but the meditations helped me approach the issues and, in a way, resolve them. I'm not quite sure how I feel about these things yet, but I feel much better about them now - it was just a week of feeling particularly down. The meditation tape did not help here, suggesting different difficulties before I'd even defined the first, let alone resolved/concluded it! But overall, some pain for a week or so was easily worth it for the last few weeks of feeling much safer/more confident, but some warning would be nice. No long term harm. |
| Doing meditations before bedtime cleared my mind such that I started dreaming again - first time I have had memorable dreams in many years. Not all of the dreams were good, however, so it sometimes felt like it would be easier not to practice. The act of thinking about my thoughts made me realise some underlying things i was worrying about. Probably positive overall, but felt negative at the time! |
| During a practice entitled 'Exploring Difficulty', where the goal was to set certain difficulties on a workbench of the mind, I found it difficult to entertain these thoughts without their taking complete hold of my mind. The practice elicited strong, unpleasant, physical and mental reactions, which I found hard to control - and which prompted me to avoid this home practice on the week it was set. I felt either that my approach, or the exercise itself, was not helpful to me at the time. |
| During the Befriending meditation, we are asked to bring someone we care about to mind. I quickly went through my family and close friends and found I had no obvious reaction or feeling when bringing these people to mind. Then, I thought of my grandma who died almost 10 years ago and started crying. This was not a problem in itself, but then I started to wonder why I didn't feel any affection for the people who are alive now and what this means for my current relationships, which I didn't find very pleasant and troubled me somewhat. |
| Feeling that meditation does not have a positive effect that I was expecting, that my progress is almost impossible to measure, feeling obliged to do the practice, being annoyed by unnecessary suggestions from the record that interrupted my flow, concentrating on pain (however, I learnt how to breathe through it, which was good) |
| Felt a bit overwhelming at times |
| Felt more emotional at certain times during the term and down, but this may have been a combination of work and mindfulness rather than purely from the course. |
| Focus with regards to thinking about other pending activities |
| I became aware of some back pain that my body had previously attenuated. It's actually quite painful. As I have become more mindful of the pain, I made adjustments to how I sleep and my posture at other times. I still have some pain, but on the whole it seems to be improving. |
| I could not linked my emotions to the course because since I started my master at Oxford I have been struggling with different emotions. But, following the questions, I realize now that I kept avoiding the home practice |
| I did not manage my time well to finish the work before the deadline |
| I don't think it was a bad thing. Spending more time thinking about my state of mind and mental health led me to seriously question some aspects of my life and this led to distress and confusion, but overall this resulted in changes that have been really beneficial. |
| I had a particularly hard week independent of the course, which coincided with the week in which we were practicing looking at difficulties, and I struggled with the practices in that week because they drove me deeper into my negative spiral of emotions. After the difficulties of that week were resolved, however, the negative aspects of the course also went away. |
| I had flu during the course - focusing on how rubbish I felt physically made me agitated and anxious |
| I have come to realise that I have very low self-confidence and do not think that I can be loved by others, which has been quite painful. I realised this during the befriending meditation and that was very, very hard. I was not able to do this practice much because of the intensity of the feelings that came with it. I also realised that I do not let myself feel much when I judge that my emotions are 'silly' or irrational: the exploring difficulties meditation was also really difficult in that respect, it made me realise this. |
| I often was very sleepy during the practices. That was one of the reasons why I did not want to do them that often. |
| I started to question the way I thought and approached things a lot more. I include this as unpleasant only because it is slightly unsettling to become aware of one's habits and thinking patterns, and creates some uncertainty. There were a few situations where I felt more self-conscious than usual, probably because I was questioning myself more and having thoughts about my experiences ("am I responding to this situation in the right way?"; "are my emotions justified?"), and that meant that I was questioning both my negative and positive reactions to events. At the same time I think it is better to become more aware of one's experiences, even if it provokes some initial uncertainty, so although slightly unsettling I would describe these experiences as overall valuable. |
| I usually use an audiobook to distract myself from thoughts that make me feel most depressed. Mindfulness gave space to these thoughts and I ended up being constantly reminded of the ways in which I could be a failure. |
| I was going through a difficult point in a relationship of mine and found myself dwelling on what had happened; which was unpleasant in itself, however, the mindful practice of sitting with it didn't make it worse than it already was. It merely reminded me of its presence and I think overall was helpful in helping me come to terms with it and deal with it in a way that I am happy with |
| I would very often get sleepy. I would also occasionally get frustrated, most having to do the practice itself when I didn't feel like it, rather than the feelings it brought up |
| I'm not sure they were really related to the course or the term in general, sleepiness most prevalent |
| Loss of concentration, aching in back, tightness in stomach |
| Mostly I became aware of examples of treating myself without kindness, which I wouldn't have thought of that way before. To a certain extent I approached difficult relationships I had with people which I would have otherwise ignored, and that wasn't always pleasant. But on the whole I found that decision to approach those relationships actually reduced the amount I tended to dwell on them. |
| no |
| occasional uncertainty when trying to see thoughts as mental events (even though I find this very beneficial and one of the most important insights overall); some physical pain in the neck and shoulders; facing difficulties sometimes felt like it was too much to take and I felt overwhelmed |
| Often felt sleepy - but this could be quite good if I was practicing in the evening as I have had trouble sleeping, and mindfulness actually helped with this. The extending kindness practice sometimes gave me feelings of guilt or regret. But this wasn't a bad experience - I tried to turn towards these feelings and observe them. More generally there was sometimes guilt if I didn't do the practice one day, or stress that I wouldn't have time for it and therefore not get the most out of the course. But I have tried to reassure myself that it is not a case of doing 'well', just learning about myself, and all these feelings come into that learning. |
| panic attack in the first session |
| realising that I was anxious, distracted and dissatisfied |
| See my previous long-text response but it was a cycle of being self-critical that I was doing something wrong because I was getting sleepy and wandering and never put that practice at the right time of day to do it properly. |
| Simply bringing awareness to how much time I waste and the ways I push away emotions or stress (to do with studying). But I feel like I always have to do whatever I need just to get through the term, so didn't feel organised enough to implement big changes. |
| Sleepiness - I wouldn't necessarily describe it as unpleasant, but it did interfere with the completion of the practice on several occasions, especially during the body scan meditation. |
| Sleepiness and sometimes being aware of the emotions felt a bit too intense. However, slowing down and taking a breathing space helped with feeling more settled afterwards. |
| Sleepiness very often. Especially with certain practices that I found more difficult (like exploring difficult thoughts) Agitation and frustration in terms of struggling to concentrate and focus sometimes, trying for a short part of it but frustrated that it didn’t work/it would end up being a waste of time because it didn’t seem to be working |
| Sleepiness, exploring difficulty there was a moment when I was doing the practice and a bad thought came to mind which was hard to let go |
| Sleepiness was a bit one Some anxiety as well |
| Sometimes had to think about things that I'd rather not, make me feel very uncomfortable and sad |
| Sometimes I was unable to calmly disconnect from stress I had prior to the session, and so when entering a focused state, sometimes I was unable to do anything but focus more on the stress I had |
| Sometimes I would just think about sad things during a meditation but I usually managed to ground myself through breathing |
| Stress and worry sometimes intensified as if I held a magnifying glass over it, but then the mindfulness practice gradually taught me to be open to this close encounter in a fruitful confrontation, rather than a crushing battle. |
| Stress related to memories that arose |
| That if mindfulness didn't work I would exhaust my options for dealing with my anxiety |
| The "Confronting Difficulty" meditation brought to mind an issue that had been weighing on my mind a lot in a way that I didn't feel was promoting my wellbeing. |
| the facing difficulty week was particularly difficult. it brought up memories and feelings that were unpleasant and for which I have no closure. thus, it was very frustrating having to go back in the wheel and think about things I've thought about before, and reach the same conclusions of hopelessness. |
| Usually this was in the form of sleepiness or distraction which was more frustrating than unpleasant, in leading to a feeling of not fully engaging with the practise. |

**Supplementary Material S5:** Participant reports of unpleasant experiences in Study 2, Time 2

| - |
| --- |
| Bad thoughts that sometimes arise or things other people say. |
| becoming aware of your thoughts can be overwhelming/ claustrophobic when you haven't been checking in with yourself, and when you do you realise how sad you have been feeling. |
| being back in the situation, feeling panicky the same way |
| Certain interactions with people were frustrating. During mindfulness these feelings arose. I was able to acknowledge them and let them go. |
| Concentrating on how I am feeling can make me realize how stressed I actually am, instead of pushing it in a corner, which can be upsetting |
| cystitis pain making me want to scream |
| During the "exploring difficulty" I sometimes got quite upset thinking about the difficulties I'm going through. For twice during my own practice I started crying. But this sad mood did not persist. |
| exploring difficulty occasionally brought up bad memories |
| Exploring difficulty was a confronting exercise as it brought me in touch with some of the stressors and anxieties that I tend to avoid or wish not to face. |
| Feeling upset, emotional, sleepy. |
| Feelings of hopelessness sometimes or of being trapped normally once a day and almost always because I am hungry, I just rarely realise it at the time. My appetite doesn't really coincide with mealtimes and I illogically won't snack because it is bad for me even when it blatantly isn't. I notice that my thoughts are unpleasant but mindfulness practice doesn't usually help because (I think) the root cause is low blood sugar but I feel at those times that eating is simply comfort eating and to be avoided… I'm not sure that my answer particularly connects with the question or the mindfulness course, sorry! The other unpleasant experience is thinking too much to go to sleep but that is nothing new, the thoughts have just changed to be ones which I find harder to dismiss. I find it far easier to dismiss emotional thoughts than I do ones about science which I don't think has made me a better person. I worry people don't like me and that I am unemotional! I have interpreted the mindfulness attitude as dismissal of feelings which I realise as I write this is completely wrong but I really don't understand. I struggle most days to fit my unrequited love into the hotcross bun model, it is a combination of those four factors but it is very very very hard to simply dismiss. I sort of only did mindfulness because the person I like did it and it helped her a lot. I therefore very strongly connect mindfulness with thoughts of her and whilst I can dismiss those thoughts during a practice I find it very draining. A study of why people embark on mindfulness courses would be very interesting. I answer that mindfulness has been quite a bit harmful in the next question which is true but it has helped in other ways. You were probably expecting one sentence here, sorry! I suppose more data doesn't hurt! |
| Finding it difficult to sleep is frustrating, and this frustration is not production so I get even more frustrated |
| I find the befriending meditation particularly difficult, I find it very difficult trying to wish myself well and this difficulty makes me extremely upset as I don't like how difficult it is just to wish myself well. I also regularly experience sleepiness when doing the meditation. |
| I found I was stressed and took longer to get to sleep due to preparing to run a marathon and submit my dissertation in the same week. I was aware I needed to sleep to be at my best for both and the pressure to sleep well often meant I didn't get to sleep very quickly. I also found thinking about the marathon made my heart race as I felt worried about what pace to run at and all the things that could go wrong, etc. |
| I have found particularly feelings that I had previously shut myself off from or rather shut down when they have cropped up difficult to deal with. It has been helpful however to bring a kinder voice to a lot of these negative feelings. So, overall the experience has been positive as it has allowed me to change my reaction to negative feelings and insecurities. |
| I have realised throughout the course that I did not let myself feel unpleasant things, so doing mindfulness has been painful at times because it has forced me to feel what I didn't let myself feel. Whilst doing the difficulty meditation, I sometimes found myself experiencing quite strong emotions, which I hadn't realised existed. The 'Wishing yourself well' meditation was also quite painful - I found it (and still do) find it very difficult to wish myself well. It has been very enlightening, however, to realise that I had these thought patterns and behaviours, as it has helped me to acknowledge that certain things were indeed painful, and that I may have problems I would have ignored before. |
| I have unpleasant thoughts regarding a relationship that ended recently. This was the focus of my meditations during the course, or rather this was the "difficulty" that I forced myself to confront. |
| I sometimes drift off into thinking about topics I've identified as intrusive during certain practices and fail to notice for a while because it feels like part of the meditation. I also often feel drowsy during practice which prevents me from getting everything possible out of it. Finally, I'm sometimes disturbed by family and friends when meditating and it can be difficult to communicate with them that I don't wish to be disturbed without 1) bringing myself out of the meditative mind-state or 2) coming across as rude. |
| I was dealing with an issue I was trying to get off my mind, and during the "confronting difficulty" meditations I found myself getting increasingly stressed about it. |
| if I was already in pain then focusing on it would make the sensation more intense. if it was a headache then it might abate after a bit, but other pain that I was coping with was increased |
| In situations of stress, I try to focus my attention in the body but sometimes I just can't and it makes me even more agitated and stressed |
| It's very frustrating becoming aware of when your thoughts are storming away and getting stuck in those thought cycles over and over again |
| Just general frustrations about not being able to focus and being much more aware of aches and pains in my back. |
| just some bad images/memories coming back up |
| Mostly high stress and anxiety related to study, exams and thinking about next steps. |
| Mostly sleepiness, which is likely reflective of how busy I am. Once in a blue moon I will have an unpleasant thought surface, usually related to something I am worrying about in general- but I am able to disassociate those from myself |
| Mostly the unpleasant feelings were agitation, sometimes fear that mindfulness wasn't going to work |
| Negative thoughts, recurring thoughts about things I need to do, ache in back and spotlight on injury in my feet |
| no |
| Not being able to concentrate, feeling irritated, fidgety |
| not related to the course, related to grief - distress and flashbacks of situation surrounding my friends' deaths last summer during the course it was worse because I was forced to be alone with my thoughts, but outside of mindfulness practice it tends to be less of an issue as I distract myself with something physical/grounding/exterior to my body |
| Occasional relationship conflicts or stress over school deadlines |
| Only really when taking a step back from experiences that I perceived to be okay, then to realise they were difficult for someone else when I look at them from a different perspective |
| Panic attacks and difficulty breathing |
| Sensation of not being socially adequate, of not fitting. Also thoughts of not being intellectually capable. |
| sleepiness. is hard to tell or distinguish what effects have been linked to the course because in the meantime mental health has been worsening |
| Sometimes irritation at myself for not doing mindfulness, feelings of having failed or not being good enough. These latter feelings if I don't feel I've sustained concentration/mindfulness throughout a meditation too Generally have not done the 'approaching difficulties' meditations since the course so have not felt any of the guilt or anything that they sometimes brought up. Nothing too unpleasant overall though |
| Sometimes when I try to actively turn towards a difficulty, I get overwhelmed by the difficulty I am trying to face, which causes upset. It is not a very strong upset or pain, but I find myself re-feeling the unpleasant feelings caused by the difficulty in a slightly more neutral way. Whenever I get overwhelmed by a difficulty I try to focus again on what we learned in the mindfulness class. I think, that with more training I will be able to improve my facing towards difficulties. |
| Stress related to coursework - came to the surface during practice |
| Tension in shoulders/neck. The mind would try shy away from the thought but over time it got easier In all fairness though, it wasn't ever that unpleasant to begin with. |
| The thoughts and difficulties meditations brought up some difficulties relating to medical issues in our family that I had mostly forgotten about until doing the course. Also found my mind was 'thinking' more about existential problems and things that were not related to what I was trying to do that that particular time |
| uncertainty about certain things in my life. questioning emotions i'd never previously explored |
| Vivid dreams, becoming aware of my biggest fear (losing my mother) |

**Supplementary Material S6:** Participant reports of harm in Study 2 Time 1

| I feel like the money I spent on it was wasted, as I didn't attend the whole course and it created extra stress for me when I avoided going |
| --- |
| It made me stress a bit more, but again, may have been due to external reasons during term. Occasionally it was helpful, but I'm not sure whether overall it has made a positive impact or not. |

**Supplementary Material S7:** Participant reports of harm in Study 2 Time 2

| Having to deal with the 'buried' issues which surfaced during the course whilst writing my thesis actually increased my stress levels, and resulted in me having to go home for a few days to unwind. However, after starting to deal with some of these issues, I think it will be overall positive. |
| --- |
| I've had thoughts that it's better not to have to pay attention to, paying them attention at the present time would be debilitating and make it hard to function in everyday life |
| My monologue in the previous section covers most of it: - expectation of a cure which simply wasn't met, - attempting to dismiss my thoughts mindfully often draws me further into them than simply ignoring them, which may be unhealthy but at least they don't change - I really don't understand how unrequited love fits into the whole model. I understand that it is unhealthy and now feel even more pressure to try to dismiss it than I did before. - Feeling like an over-rational robot, which I might have been before but I quite happily didn't realise. - Spending a lot of time trying to work out how to escape from society whilst also feeling lonely, which admittedly I did do before but less so. In conclusion - the slightly and moderately distressing thoughts are easy to dismiss which has left me with inly the big ones. The mindfulness tape in which you a supposed to address these thoughts (I feel) really piles into you, asking you to drag up more and more thoughts which can be incredibly overwhelming. It is that tape which has put me off listening to any of them at all and really loathing the patronising narrator. |
| somewhat' is probably an exaggeration - throughout the mindfulness course I was still in shock regarding my friend's suicide (though I didn't realise that at the time) and it was too soon for me to do the course - I had a panic attack in the first session and then often did not try participate in practices during the subsequent sessions out of expecting the same thing to happen at some points it made me aware of how unhappy/distressed I still was due to grief/shock, which I don't think was particularly useful to address at that point in time, so in that way it was harmful, although there has been no long term harm whatsoever. |
